# PD71 Survival Outcomes And Adherence To Defined Daily Doses Of Imiglucerase: A 16-Year Brazilian Cohort Study In Gaucher Disease

**DOI:** 10.1017/S0266462324003283

**Published:** 2025-01-07

**Authors:** Marcus Carvalho Borin, Francisco de Assis Acurcio, Juliana Alvares-Teodoro, Augusto Guerra

## Abstract

**Introduction:**

Gaucher disease is characterized by a deficiency of the enzyme glucocerebrosidase and requires lifelong enzyme replacement therapy. Imiglucerase is the standard treatment, which improves patient survival and quality of life. While defined daily doses (DDD) offer a standardized metric, the relative efficacy of adhering strictly to these guidelines, compared with tailored lower doses, has not been fully explored.

**Methods:**

A retrospective cohort study was conducted on 1,234 patients to investigate the survival outcomes associated with various levels of adherence to DDDs of imiglucerase, factoring in demographic diversity and comorbidity profiles, and to evaluate the feasibility of a more personalized dosing approach in the management of Gaucher disease. DDD adherence was categorized as equal to DDD, higher than DDD, or lower than DDD. Kaplan-Meier survival analysis, log-rank tests, and Cox proportional hazards models were used to assess survival probabilities over 16 years. Data on age, sex, comorbidities, and other demographic factors were collected to adjust for potential confounders.

**Results:**

Over the 16-year period, our Kaplan-Meier survival analysis revealed distinct survival probabilities across the three groups based on their adherence to DDD of imiglucerase. Patients who received doses lower than DDD (n=880) had a survival probability of 91.8 percent. In contrast, those receiving doses equal to the DDD (n=15) had a 100 percent survival probability, since no events were observed in this group. The greater than DDD group (n=339) exhibited a survival probability of 81 percent. A log-rank test indicated a borderline statistical significance (p=0.058) in the survival distributions among the various DDD adherence levels, with a favorable trend in the lower dose group.

**Conclusions:**

Our research indicates that lower than usual doses of imiglucerase may improve survival rates in patients with Gaucher disease. This finding suggests that reduced dosages could lead to better clinical outcomes with fewer side effects, highlighting the potential benefits of personalized dosing strategies. Further studies are needed to confirm these preliminary results and optimize dosing protocols.

